# Validation of the Malay Version of the Shame and Stigma Scale among Cancer Patients in Malaysia

**DOI:** 10.3390/ijerph192114266

**Published:** 2022-11-01

**Authors:** Zheng Zhang, Nizuwan Azman, Hui Ting Eyu, Nik Ruzyanei Nik Jaafar, Hajar Mohd Salleh Sahimi, Mohd Razif Mohamad Yunus, Noorsuzana Mohd Shariff, Rohayu Hami, Nor Shuhada Mansor, Ping Lu, Mohammad Farris Iman Leong Bin Abdullah

**Affiliations:** 1 Department of Oncology, 1st Affiliated Hospital, Xinxiang Medical University, Xinxiang 453100, China; 2 Department of Community Health, Advanced Medical and Dental Institute, Universiti Sains Malaysia, Kepala Batas 13200, Malaysia; 3 Division of Research and Networking, Advanced Medical and Dental Institute, Universiti Sains Malaysia, Kepala Batas 13200, Malaysia; 4 Department of Psychiatry, Faculty of Medicine, Universiti Kebangsaan Malaysia, Cheras 56000, Malaysia; 5 Department of Otorhinolaryngology, Faculty of Medicine, Universiti Kebangsaan Malaysia, Cheras 56000, Malaysia

**Keywords:** stigma, cancer patients, Malaysia, Malay version of the Shame and Stigma Scale, reliability, validity

## Abstract

The assessment of stigma among cancer patients is of the utmost importance as stigma may lead to various psychological sequelae and a lower quality of life. This study aimed to translate the English version of the Shame and Stigma Scale (SSS) into Malay and validate the Malay version of the SSS (SSS-M) to assess the degree of stigma among cancer patients in Malaysia. Initially, the concurrent translation and back translation of the SSS-M were performed, and the face and content validity were assessed. Subsequently, the SSS-M was administered to a total of 234 patients with mixed types of cancer to assess its reliability (internal consistency and test–retest reliability), construct validity (convergent and discriminant validity), and conduct an exploratory factor analysis (EFA) and a confirmatory factor analysis (CFA). The SSS-M total score registered a good internal consistency (a Cronbach’s α of 0.881) and test–retest reliability (an intraclass correlation coefficient of 0.876, *p* < 0.001). The EFA and CFA confirmed that the SSS-M consisted of 16 items in 3 domains. Its convergent and discriminant validity were achieved. Hence, the SSS-M demonstrated good psychometric properties and is available for use to assess stigma among cancer patients in Malaysia.

## 1. Introduction

As the prevalence of cancer continues to rise over time, it has become a major public health concern globally. According to the World Cancer Report from the World Health Organization (WHO) there were 19.29 million new cancer cases worldwide, and breast cancer has replaced lung cancer as the world’s most common cancer diagnosis in 2020 [1]. Cancer is the fourth leading cause of death among adolescents and young adults worldwide [2]. Similarly, in Malaysia, the incidence of cancer is increasing over time. The Malaysia National cancer registry report (MNCR) revealed that a total of 115,238 new cancer cases were diagnosed between 2012–2016 which represented an 11% increase in new cancer cases and a 30% increase in mortality compared to the 2007–2011 report, and the most common cancer reported was breast cancer [3]. 

Cancer patients often suffer from psychological distress which not only affects the treatment of cancer and quality of life (QoL), but also is being regarded as an independent risk factor for increased cancer mortality [4]. Stigma refers to an inner shame experience of patients due to having the disease and is regarded as a negative psychological stress response [5]. Patients who suffer from public avoidance and exclusion in social interactions are prone to public stigma. In essence, patients who have been discriminated against for a long time will develop self-doubt and shame leading to the development of self-stigma [6]. 

A meta-analysis which included a total of 7114 cancer patients indicated that cancer- related stigma induced anxiety, depression, a lower QoL and stressful life events [7]. A high degree of stigma was reported among breast and cervical cancer patients [8]. Meacham et al. (2016) showed that the stigma associated with breast cancer had an influence on treatment and care engagement [9]. While in head and neck cancer patients, the stigma greatly affected psychological well-being [10], and it was significantly correlated with negative psychological consequences, especially in those who suffered from severe facial disfigurement [11]. In addition, medical help-seeking was found to be independently related to stigma in lung cancer patients [12]. 

Despite the importance of screening for stigma among cancer patients, as stigma increases the risk of psychological sequelae and affects QoL, data on stigma among cancer patients in Malaysia is lacking. Hence, there is a need to adapt and validate an instrument for assessing the degree of stigma among patients with various types of cancer in Malaysia. There are validated screening tools used to measure stigma against cancer. The Social Impact Scale is used to assess stigma in people with AIDS or cancer and it is non-specific [13]. The Cataldo lung cancer stigma scale is a multidimensional measurement tool adapted from the HIV Stigma Scale to measure stigma among lung cancer patients, but it consists of 31 items which require a long duration of administration [14]. The Lung Cancer Stigma Inventory was developed by Hamann et al. (2018) to evaluate the lung cancer stigma and consists of 25 items [15], while the Shame and Stigma Scale (SSS) measures the degree of stigma among head and neck cancer patients [16]. The SSS contains four domains and twenty items: shame with appearance (8 items), sense of stigma (6 items), regrets (3 items) and social concerns (3 items). Therefore, it has a relatively shorter time of administration, which is suitable for use to assess stigma among cancer patients as their concentration to answer a questionnaire may be affected by the symptoms of the illness and/or the adverse effects of treatment. The Cronbach α of the SSS was 0.94, and the Cronbach α of each domain ranged from 0.78 to 0.90, indicating a good to excellent internal consistency [16]. To date, the SSS has been translated and validated in Portuguese with a Cronbach α of 0.85 [17], in Chinese with a Cronbach α of 0.85 [18], and in Hindi with a Cronbach α of 0.85 [19]. However, the SSS has not been translated into the Malay language, or adapted and validated for use to assess stigmas among patients with all types of cancer in Malaysia. Hence, in this study, we translated the original English version of the SSS into Malay, assessed the psychometric properties of the Malay version of the SSS (SSS-M), such as internal consistency and test–retest reliability, face, content and construct validity (convergent and discriminant validity), as well as performed exploratory and confirmatory factor analyses to confirm its domain structures for its adaptation to be used for assessing the degree of stigma among cancer patients in Malaysia. 

## 2. Materials and Methods

### 2.1. Study Design

This study received approval from the Human Research Ethics Committee of Universiti Sains Malaysia (code: USM/JEPeM/21040321) and the Research Ethics Committee of Universiti Kebangsaan Malaysia (code: UKM/PPI/111/8/JEP-2021-753) and abides by the regulations of the 1964 Declaration of Helsinki and its amendments. This validation study was conducted between January 2022 to July 2022 whereby the source population was cancer patients who were registered at the Advanced Medical and Dental Institute (AMDI), Universiti Sains Malaysia (USM) and Universiti Kebangsaan Malaysia Medical Centre (UKMMC). AMDI, USM is a tertiary referral center for cancer patients in the Northern region of Peninsular Malaysia, while UKMMC is a tertiary referral center for cancer patients and a teaching hospital in the central region of Peninsular Malaysia. Hence, the selection of these two medical centers will cover the cancer populations in the northern and central regions of Peninsular Malaysia. The sample size calculation was performed as follows:

(a) Calculation of sample size for internal consistency was performed using the Statstodo Programme where the probability of type I error = 0.05, power = 1 − β = 0.8, expected Cronbach’s α = 0.95 [20], sample size required for each item = 4 subjects, and total number of items = 20 items. Hence, the estimated sample size required was 80 subjects;
(b) Calculation of sample size for test–retest reliability was performed using the G*Power 3.1.9.7 sample size calculator, whereby the probability of type I error = 0.05, power = 1 − β = 0.8, and H1 corr ρ_ac = −0.2. Hence, the estimated sample size required was 192 subjects;
(c) Calculation of sample size for exploratory and confirmatory factor analysis:
  (i) For calculation of sample size for the exploratory factor analysis of the SSS-M, we refer to the Rule of 5 which states that the sample size for an exploratory factor analysis should be at least five times the number of observed variables to be studied [21]. Hence, the subjects-to-variables ratio should be at least five. The total number of items in the two questionnaires was 20. Therefore, the estimated sample size was 100 subjects.
  (ii) For calculation of sample size for the confirmatory factor analysis for SSS-M, we referred to the validation of SSS study by Kissane et al. (2013) [16] and calculated the estimated sample size using the A-priori Sample Size calculator for Structural Equation Models. The effect size was 0.25, power at 0.8, number of latent variables was 4, number of observed variables was 20, and probability of type I error = 0.05. Hence, the estimated sample size needed was 175.

Consequently, based on all three calculations, the largest sample size required was 192 subjects. Hence, the sample size needed for the validation of the Malay version of the SSS was 230 subjects (inclusive of a 20% drop out rate). 

The participants in this study were recruited via consecutive sampling. Initially, cancer patients who attended the oncology clinics of AMDI, USM and UKMMC were approached by the research assistant and screened for the inclusion and exclusion criteria. The inclusion criteria included those who were: (a) diagnosed with any type of cancer and at any stage of cancer, (b) able to read and write in Malay language, and (c) age 18 years and above. While the exclusion criterion was: (a) those with a history of mental illness and other medical illnesses. Those who fulfilled all the inclusion criteria and with no exclusion criterion were approached by the research team and informed about the study, including the purpose and description of the study procedures, risks and benefits, the subject’s right to withdraw from the study and assured anonymity of the data collected before they signed the informed consent to participate in the study. 

### 2.2. Translation and Back Translation of the SSS-M and Content Validity

Initially, the original English version of the SSS was translated into the Malay language by a bilingual language expert who is a native speaker of Malay and a bilingual native Malay speaker in the research team. Neither of the translators were in contact with each other. Subsequently, the two translators discussed the translated copies of the questionnaires to construct a third jointly translated copy of the Malay versions of both the questionnaires. Similarly, a bilingual language expert who is a native speaker of English and had not seen the original English version of the questionnaires back translated the draft of the Malay version of the questionnaire into English. Subsequently, all the translators discussed the translated and back-translated copies of the questionnaires with the research project coordinator to construct the harmonized copies of the translated and back-translated questionnaires. 

Subsequently, these translated and back-translated harmonized copies of the questionnaire were examined by a team of content experts consisting of an oncologist, a psychiatrist, two psychologists, and two community health specialists to construct the first draft of the Malay version of the Stigma and Shame Scale (SSS-M). Each expert was asked to assess the relevance of the questions and the response options for each item of the SSS-M. The rating options by the experts were as follows: “item is not relevant to the measured domains”, “item is relevant to the measured domain” and “item is very relevant to the measured domain”. Experts who rated the item as “item is not relevant to the measured domains” were given a score of 0, while experts who rated the item as “item is relevant to the measured domain” and “item is very relevant to the measured domain” were given a score of 1. The item-level content validity index (I-CVI) for each item was measured as the number of experts who gave a rating of “relevant” and “very relevant” for the item relative to their measured domain divided by the total number of experts. A value of ≥0.83 was considered acceptable. The scale-level content validity index according to the universal agreement (UA) among experts (S-CVI/UA) was evaluated as the sum of all the items in the SSS-M with a UA of equal to 1, divided by the total number of items of the SSS-M. The UA for an item was scored as 0 if not all the experts rated the item as “relevant” or “very relevant” to the measured domain, while the UA was scored as 1 if all the experts rated the item as “relevant” or “very relevant” to the measured domain. The average scale-level CVI (S-CVI/Ave) was assessed as the sum of the I-CVI divided by the total number of items in the SSS-M. A S-CVI/UA score of >0.8 and a S-CVI/Ave score of >0.9 were considered as having a high CVI. 

Following this, the draft of the SSS-M was administered to 20 native Malay speaking cancer patients recruited from AMDI, USM to assess the semantic quality, comprehensibility and appropriateness of the administration duration. They were interviewed to pinpoint any redundant sentences, the wording and to assess the time taken to complete the questionnaires. They were asked to rate whether the words, sentences and instructions of the SSS-M were “not appropriate”, “appropriate” or “very appropriate” and to comment on any wording and sentences which need to be amended. In the pilot study, 65% of the respondents rated the semantic quality, comprehensibility and appropriateness of the administration duration of all the wordings, sentences and instructions of the SSS-M as “appropriate” and another 35% rated the SSS-M as “very appropriate”. There were no comments on any redundant wordings and sentences and no need to amend any wordings, sentences, and instructions. Hence, the SSS-M did not require further amendment from the panel of experts. 

### 2.3. Measures

Initially, during the baseline assessment, 234 cancer patients were recruited and given the socio–demographic and clinical characteristics questionnaire and the SSS-M. Subsequently, a follow up assessment commenced 3 weeks after the baseline assessment and the same participants were re-assessed with the SSS-M to evaluate the test–retest reliability. There were 117 participants who completed the follow-up assessment. 

The socio–demographic and clinical characteristics questionnaire included data on age, gender, ethnicity, monthly household income, marital status, education level, types of cancer and stage of cancer. Each participant’s age could be reported as “18–25 years old”, “26–45 years old”, “46–65 years old” and “more than 65 years”. Gender could be registered as “male” and “female”. Ethnicity could be recorded as “Malay”, “Chinese” and “Indian”. Monthly household income could be documented as “less than RM 4500”, “RM 4500-RM 11000” and “more than RM 11000”. The marital status of the participants could be recorded as “married” and “single/divorcee/widow/widower”. The education level of the participants could be reported as “up to primary education or below”, “up to secondary education” and “up to tertiary education”. As for the types of cancer, they could be documented as “breast cancer”, “head and neck cancer”, “colorectal cancer” and “other types of cancer”. Finally, the stage of cancer could be documented as “stage 1”, “stage 2”, “stage 3” and “stage 4”. The data on the clinical characteristics of the participants were initially provided by the participants and the validity was confirmed by counterchecking the data with their medical files. 

The Shame and Stigma Scale (SSS) is a self-administered instrument for evaluating the sense of shame and stigma reported by head and neck cancer patients. It is a self-reported instrument consisting of 20 items, designated into four domains. Eight items are assigned to shame and appearance, three are assigned to social isolation, six to the feeling of stigma, and three items to regrets. Each item is scored in a 5-point Likert scale ranging from 0 to 4, where 0 indicates ‘‘never’’, 1 indicates ‘‘seldom’’, 2 depicts ‘‘sometimes’’, 3 depicts ‘‘often’’ and 4 corresponds to ‘‘all the time’’. Hence, its total score could range from 0 to 80, where a higher score indicated a higher degree of stigma due to cancer [16].

### 2.4. Statistical Analysis

All the data was analyzed by the Statistical Package for Social Sciences version 26 (SPSS 26; SPSS Inc., Chicago, IL, USA), except for the confirmatory factor analysis, which was performed using the SPSS Amos version 26 software (SPSS Amos 26). Descriptive statistics for the socio–demographic and clinical characteristics, and the SSS-M scores at the baseline and follow-up were presented. All nominal data were presented as frequency and percentage, while all continuous data were presented as the mean and standard deviation (SD). The internal consistency of all the domains and the total score of SSS-M (in Cronbach’s α) were assessed to measure the reliability of the SSS-M. A Cronbach’s α of >0.7 was considered as acceptable. The test–retest reliability was also computed to further measure the reliability of the SSS-M, which was presented as an intraclass correlation coefficient (ICC). The ICC of >0.5 indicated an acceptable reliability, while a value of 0.75 to 0.90 depicted a good reliability, and a value of >0.90 indicated an excellent reliability. In addition, the composite reliability index (CR index) was also calculated for all the domains and the total score of the SSS-M by using the factor loading of items obtained from the confirmatory factor analysis (CFA), whereby a value of 0.8 and above was considered as acceptable [22].

The construct validity of the SSS-M was evaluated with the exploratory factor analysis (EFA), CFA, and the convergent and discriminant validity. To test for the normality of the factors, the skewness and kurtosis were computed. The skewness (s) and kurtosis (k) of the items in SSS-M are as follows: shame with appearance (s = 0.452, k = 2.606), sense of stigma (s = 0.344, k = 2.568), regret (s = 0.396, k = 2.439) and total SSS-M (s = 0.443, k = 2.557), indicating that they were normally distributed. In the EFA assessment, the factor extraction was initially performed using a parallel analysis for the SSS-M, whereby the Eigenvalue of the extracted factors generated from maximum likelihood estimation was compared with the mean Eigenvalue generated from the parallel analysis. The number of factors extracted was determined by the number of factors which had a mean Eigenvalue of less than that of the respective Eigenvalue in the maximum likelihood estimation. The Kaiser–Meyer–Olkin measure of sample adequacy value of >0.6 was considered as acceptable; the Barlett’s test of sphericity in which a *p*-value of <0.05 indicated a valid EFA. The factor extraction was followed by the Promax oblique rotation of variables, by which only the items with a factor loading of >0.4 were considered acceptable. In the CFA assessment, the best fitting factor model of the SSS-M was determined based on several variables: (a) a chi-square whereby a non-significant *p*-value (*p* > 0.05) was considered acceptable, (b) a chi-square/degree of freedom (ꭓ^2^/df) of <3.0 was considered acceptable, (c) a Tucker–Lewis index (TLI) of ≥0.95 was considered acceptable, (d) a comparative fit index (CFI) of ≥0.95 was considered acceptable, (e) a goodness of fit index (GFI) of ≥0.90 was considered acceptable and (f) a root mean square error of approximation (RMSEA) of <0.06 was taken as acceptable. The convergent validity of the SSS-M was evaluated by referring to the best fitting factor model of the SSS-M confirmed by CFA, by which the average variance extracted (AVE) was calculated as the (sum of the squared factor loadings of the items designated to the measured domain)/(sum of the total number of indicators). An AVE of >0.5 was taken as acceptable indicating that the SSS-M had achieved convergent validity. As for discriminant validity, it was again assessed based on the CFA’s best fitting factor model of the SSS-M, whereby if the square root of AVE of the measured domain was higher than all the inter-construct correlations between the domains of the SSS-M, the discriminant validity was considered to be achieved. 

## 3. Results

### 3.1. Participants 

The socio–demographic and clinical characteristics, and the total SSS-M scores of all the participants are presented in Table 1. More than half of the participants were females (*n* = 153, 65.4%) and slightly more than half of them were within the middle age group that was between 46 to 65 years old (*n* = 120, 52.3%). The majority of the participants were within the low-income group, earning less than RM 4500 per month (*n* = 193, 82.5%). In the context of clinical characteristics, slightly more than two fifths of the participants were diagnosed with breast cancer (*n* = 100, 42.7%) and more than half of the participants were in more advanced stages of cancer (stage 3 and 4, *n* = 144, 61.5%). The mean total SSS-M score at baseline was 17.37 (SD = 11.03), whereas the mean total SSS-M score at follow-up was 18.19 (SD = 12.68). 

### 3.2. Content Validity Index of the SSS-M

The content validity index of the SSS-M is summarized in Table 2. The I-CVI of all the items in the SSS-M ranged from 0.83 to 1.0. The S-CVI/Ave of the SSS-M was 0.97. Finally, the S-CVI/UA of the SSS-M was 0.85. 

### 3.3. Exploratory and Confirmatory Factor Analyses of the SSS-M

The factor extraction in the exploratory factor analysis by comparing the Eigenvalue of factors from maximum likelihood estimation and the parallel analysis are presented in Table 3. A total of four factors were extracted (four factors with a mean Eigenvalue in the parallel analysis smaller than the Eigenvalue in the maximum likelihood estimation). We omitted the factor that comprised items 1R, 4R, 7R and 20R as these were positively worded items that resulted in an additional factor being extracted and could contribute to a method effect (this will be explained further in the Discussion section). Hence, a total of three factors were maintained. The exploratory factor analysis of the SSS-M (3-factor model) with a maximum likelihood estimation, Promax oblique rotation and Kaiser normalization is summarized in Table 4. The Kaiser–Meyer–Olkin measure of sample adequacy value of 0.892 and the Barlett’s test of sphericity were significant (*p* < 0.001). The Promax oblique rotation of the variables revealed that the shame with appearance domain consisted of five items with factor loadings ranged between 0.516 to 0.845 (items 2, 3, 5, 6 and 8), while the sense of stigma domain had eight items with factor loadings ranged between 0.482 to 0.869 (items 9, 10, 11, 12, 13, 14, 18 and 19). The regrets domain was made up of three items with factor loadings ranged from 0.479 to 0.994 (items 15, 16 and 17). All three factors contributed to a total variance of 57.336% of the SSS-M. 

Considering the CFA assessment of the SSS-M, a 4-factor model of the SSS-M whereby the items were allocated to domains similar to that of the original English version of the SSS was not fitting (χ^2^ = 317.788, *p* < 0.001, ꭓ^2^/df = 1.986, TLI = 0.889, CFI = 0.906, GFI = 0.874 and RMSEA = 0.065). Following this, a 3-factor model of the SSS-M with all 20 items of the SSS included in the model was also not fitting (χ^2^ = 318.557, *p* < 0.001, ꭓ^2^/df = 1.979, TLI = 0.889, CFI = 0.906, GFI = 0.876 and RMSEA = 0.065). Finally, a 3-factor model with the items’ allocation to the domain being similar to the EFA findings (with 16 items; whereby items 2, 3, 5, 6 and 8 were designated to the shame with appearance domain, items 9, 10, 11, 12, 13, 14, 18 and 19 were designated to the sense of stigma domain, items 15, 16 and 17 were designated to the regrets domain and items 1R, 4R, 7R and 20R were omitted) was the best-fitting model of the SSS-M (χ^2^ = 187.686, *p* < 0.001, ꭓ^2^/df = 1.896, TLI = 0.959, CFI = 0.952, GFI = 0.906 and RMSEA = 0.052). The CFA findings of the SSS-M are summarized in Table 5. 

### 3.4. The Convergent and Discriminant Validity of the SSS-M

The evaluation of the convergent and discriminant validity of the SSS-M, which was based on the best-fitting 3-factor model of the SSS-M (16 items) is presented in Table 6. The average variance extracted (AVE) of all five factors confirmed for the SSS-M ranged from 0.510 to 0.557. The square root of the AVE for the shame with appearance (SWA) domain was 0.746, which was higher than the inter-construct correlations of the SWA domain with all other domains of the SSS-M (correlations were 0.570 and 0.708). The square root of the AVE for the sense of stigma (SS) domain was 0.714, which was higher than the inter-construct correlations of the SS domain with all other domains of the SSS-M (correlations were 0.640 and 0.708). Similarly, the square root of the AVE for the regrets (R) domain was 0.719, which was higher than the inter-construct correlations of the R domain with all other domains of the SSS-M (correlations were 0.570 and 0.640). 

### 3.5. Reliability of the SSS-M (Internal Consistency, Composite Reliability and Test–Retest Reliability)

The internal consistency of the domains of the SSS-M exhibited a Cronbach’s α ranging from 0.685 to 0.854, whereas the internal consistency of the total SSS-M score registered a Cronbach’s α of 0.881. In the context of the test–retest reliability, the intraclass correlation coefficient (ICC) of the domains of the SSS-M ranged from 0.756 to 0.843 and all were statistically significant (*p* < 0.001). While the intraclass correlation coefficient of the total SSS-M score was 0.876 and statistically significant (*p* < 0.001). In addition, the composite reliability index of the total SSS-M was 0.946, while those of the domains of the SSS-M ranged from 0.758 to 0.892. The internal consistency, composite reliability index and test–retest reliability of the SSS-M are summarized in Table 7. 

## 4. Discussion

This study translated the original English version of the SSS into Malay and validated and adapted the SSS-M for assessing shame and stigma among cancer patients in Malaysia by evaluating the reliability and validity of the SSS-M among Malaysian patients with various types of cancer. The cancer patients in this study registered a degree of stigma (mean total SSS-M_baseline_ = 17.34, SD = 11.03; mean total SSS-M_follow up_ = 18.19, SD = 12.68) comparable with that of other cancer patients (mean total SSS = 16.5, SD = 16.1 in Brazilian cancer patients; mean total SSS = 18.08, SD = 14.67 in American cancer patients) [16,23]. 

In terms of reliability, the SSS-M and its domains exhibited an acceptable to good internal consistency except for the regret (a Cronbach’s α of 0.685) domain. Similarly, the composite reliability index of the SSS-M and its domains were acceptable except for the regret domain (0.758). It is common that too few items designated to the measured domain may result in both a low Cronbach’s α value and a low composite reliability index of the measured domain [22,24]—regret has only 3 items. It is also expected that the composite reliability index of the SSS-M and its domains were higher than the Cronbach’s α values [22]. Otherwise, the internal consistency of the SSS-M total score (a Cronbach’s α of 0.881) was similar to that of the Chinese version of the SSS (a Cronbach’s α of 0.85) [18] and the Hindi version of the SSS (a Cronbach’s α of 0.85) [19], as well as almost comparable to the internal consistency of the original English version of the SSS (a Cronbach’s α of 0.94) [16]. As for the test–retest reliability, the domains (the ICC ranged from 0.756 to 0.843, *p* < 0.001) and the total SSS-M (ICC = 0.876, *p* < 0.001) registered good to excellent test–retest reliability as compared to the domains (the ICC ranged from 0.295 to 0.680, *p* < 0.05) and total score (ICC = 0.655, *p* < 0.05) of the Chinese version of the SSS [18]. 

The translation and back translation of the SSS-M followed the standard procedures of translation of questionnaires by the International Test Commission [25]. In the context of the content validity index, the I-CVI of all the items in the SSS-M ranged from 0.83 to 1.00, indicating acceptable I-CVI, whereas the S-CVI/Ave was above 0.9 and the S-CVI/UA of the SSS-M was above 0.8, denoting that the content validity index was good. In the pilot study to test the semantic quality, comprehensibility and appropriateness of the administration duration of the SSS-M, the native Malay speaking cancer respondents commented that all the wordings, sentences and instructions as well as the duration of administration were either “appropriate” or “very appropriate”, with no redundant structures and no need for amendment. These findings revealed that the SSS-M had achieved a good face and content validity. 

The EFAs performed in this study extracted three factors with the factor loadings of all the items designated to their respective domains were above 0.4. However, in the SSS-M, we could not extract items which are representative of the social concern domain. Instead, we extracted a factor representing positive perception, which consisted of the items 1R, 4R, 7R and 20R. However, these positively worded items were omitted with respect to the possibility of a method effect, whereby any characteristics of the measurement by an instrument, which contribute to the variance of scores (beyond what is attributed to the construct of interest), results in a systematic variance irrelevant to the study objectives [26]. Moreover, the CFA of the SSS-M confirmed that the factor structures and model extracted by the EFA was indeed the best fitting model of the SSS-M: consisting of 3 domains with 16 items, in which items 2, 3, 5, 6 and 8 were designated to the shame of appearance domain; items 9, 10, 11, 12, 13, 14, 18 and 19 were designated to the sense of stigma domain; and items 15, 16, and 17 were designated to the regret domain. The discrepancy in the item’s allocation and factor structures of the SSS-M as compared to the original English version of the SSS may be due to differences in the language used and cultural differences. The wordings available in both languages to describe the meaning of the items of the SSS may differ, which may result in this discrepancy. In addition, the explanation of how the domain comprised of items 1R, 4R, 7R and 20R was extracted may be due to differences in the understanding of the reverse scoring items of the SSS between the participants in this study and that of the validation study of the original English version of the SSS, as pinpointed by Goyal et al. (2021) [19]. This may also contribute to a method effect contributed by these positively worded items (items 1R, 4R, 7R and 20R). Despite the chi-square of the best fitting model of the SSS-M (16 items, 3-factor model) being statistically significant (*p* < 0.001), if the sample size is large enough, then a small difference between the empirically found distribution of the items in factors and the distribution of items in factors indicated by the null hypothesis, will contribute to a statistically significant chi-square value, which is deemed acceptable [27]. Hence, the 16-item 3-factor model similar to that suggested by the EFA, which was confirmed by the CFA in this study, is indeed valid.

In terms of the convergent validity of the SSS-M, based on the best fitting 3-factor model (with 16 items) of the SSS-M confirmed by the CFA, the AVE of all the three domains of the SSS-M were more than 0.5 (Table 6), indicating that the convergent validity was achieved. As for the discriminant validity of the SSS-M, since all the square roots of AVE of all the domains of the SSS-M were higher than the inter-construct correlations of all the domains (Table 6), the discriminant validity of the SSS-M was achieved.

This validation study had a few limitations. First, the concurrent validity was not assessed in this study as there is no gold standard instrument in the translated Malay version, which evaluates stigma among cancer patients in Malaysia. Second, the gender and ethnicity distributions of the study sample were not representative of the cancer population in Malaysia. Hence, this will affect the generalizability of our research findings. 

Despite these limitations, this study successfully translated the original English version of the SSS into Malay and validated the Malay version of the SSS among cancer patients in Malaysia. Clinically, it is pivotal to screen for stigma among cancer patients, which could contribute to several psychological sequelae and lower the quality of life of cancer patients as well as provide important data to guide the development of psychosocial interventions to reduce stigma among cancer patients in Malaysia. Hence, a validated screening tool for stigma among Malaysian cancer patients, such as the SSS-M, is of the utmost importance for clinical use. 

## 5. Conclusions

The SSS-M was successfully translated from the original English version of the SSS and exhibited good psychometric properties, such as internal consistency and test–retest reliability (reliability), face, and content and construct validity (validity). The EFA and CFA confirmed that the SSS-M was made up of 16 items allocated to three domains (shame with appearance, sense of stigma and regret). The SSS-M can now be used to assess perception of shame and stigma among the cancer population in Malaysia. 

## Figures and Tables

**Table 1:** Socio–demographic and clinical characteristics of the participants.

Variables	Number of Participants	Percentage
	(*n*)	(%)
Age:		
18–25 years old	2	0.9
26–45 years	63	26.9
46–65 years	120	51.3
>65 years	49	20.9
Gender:		
Male	81	34.6
Female	153	65.4
Ethnicity:		
Malays	182	77.8
Chinese	37	15.8
Indians	15	6.4
Monthly household income:		
<RM 4500	193	82.5
RM 4500–RM 11,000	35	15
>RM 11,000	6	2.5
Marital status:		
Married	197	84.2
Single/divorcee/widow/widower	37	15.8
Education status:		
Primary education or below	31	13.2
Up to secondary education	130	55.6
Tertiary education and above	73	31.2
Types of cancer:		
Breast cancer	100	42.7
Head and neck cancer	72	30.8
Colorectal carcinoma	28	12
Others	34	14.5
Stage of cancer:		
Stage 1	26	11.1
Stage 2	64	27.4
Stage 3	78	33.3
Stage 4	66	28.2
Total SSS score (baseline)	17.37 ^a^	11.03 ^b^
Total SSS score (follow up)	18.19 ^a^	12.68 ^b^

^a^ Mean, ^b^ standard deviation.

**Table 2:** Content validity index (CVI) of the SSS-M by six experts.

Items	Expert 1	Expert 2	Expert 3	Expert 4	Expert 5	Expert 6	Experts in Agreement	I-CVI	UA
Item 1	1	1	1	1	1	1	6	1	1
Item 2	1	1	1	1	1	1	6	1	1
Item 3	1	1	1	1	1	1	6	1	1
Item 4	1	1	0	1	1	1	5	0.83	0
Item 5	1	1	1	1	1	1	6	1	1
Item 6	1	1	1	1	1	1	6	1	1
Item 7	1	1	1	1	1	1	6	1	1
Item 8	1	1	1	1	1	1	6	1	1
Item 9	1	1	1	1	1	1	6	1	1
Item 10	1	1	1	1	1	1	6	1	1
Item 11	1	1	1	1	1	1	6	1	1
Item 12	1	1	1	1	1	1	6	1	1
Item 13	1	1	1	1	1	1	6	1	1
Item 14	1	1	1	0	1	1	5	0.83	0
Item 15	1	1	1	1	1	1	6	1	1
Item 16	1	1	1	1	1	1	6	1	1
Item 17	1	1	1	0	1	1	6	0.83	0
Item 18	1	1	1	1	1	1	6	1	1
Item 19	1	1	1	1	1	1	6	1	1
Item 20	1	1	1	1	1	1	6	1	1
Proportion relevance	1.00	1.00	0.95	0.90	1.00	1.00			
Average proportion of items judged as relevant across the six experts						0.98	S-CVI/Ave:	0.97	
							S-CVI/UA:		0.85

I-CVI = item-level content validity index, UA = universal agreement, S-CVI/Ave = average of the scale-level content validity index, S-CVI/UA = average of the scale-level content validity index across universal agreement among experts.

**Table 3:** Factor extraction in the exploratory factor analysis by comparing the Eigenvalue of factors from the maximum likelihood estimation and parallel analysis.

Factor	Eigenvalue from Maximum Likelihood Factoring	Mean Eigenvalue from Parallel Analysis
1	6.516	1.480
2	1.446	1.366
3	1.213	1.190
4	1.123	1.120
5	1.003	1.061

**Table 4:** Exploratory factor analysis using the maximum likelihood estimation with Promax rotation and Kaiser normalization for the SSS-M (3-factor model).

Items	Shame with Appearance	Sense of Stigma	Regret
Item 2	**0.532**		
Item 3	**0.674**	0.463	
Item 5	**0.516**	0.408	
Item 6	**0.845**	0.566	
Item 8	**0.533**	0.441	
Item 9		**0.482**	
Item 10	0.571	**0.812**	
Item 11	0.596	**0.869**	
Item 12	0.611	**0.635**	
Item 13	0.411	**0.647**	
Item 14	0.652	**0.670**	0.425
Item 18	0.598	**0.785**	
Item 19	0.596	**0.665**	
Item 15	0.515	0.540	**0.590**
Item 16			**0.479**
Item 17			**0.994**
Eigenvalue	1.446	6.516	1.213
Variance (%)	9.035	40.722	7.579
Total variance (%)			57.336

**Table 5:** The confirmatory factor analysis of three different models of the Malay version of the Shame and Stigma Scale (SSS-M).

Variables	3-Factor Model (According to EFA, Positive Worded Items Deleted)	3-Factor Model (All 20 Items Included)	4-Factor Model (According to Original English Version of the SSS)
Chi-square (*p*-value)	187.686 (*p* < 0.001) *	318.557 (*p* < 0.001) *	317.788 (*p* < 0.001) *
Chi-square/degree of freedom (ꭓ^2^/df)	1.896	1.979	1.986
Comparative fit index (CFI)	0.952	0.906	0.906
Goodness of fit index (GFI)	0.906	0.876	0.874
Tucker–Lewis index (TLI)	0.959	0.889	0.889
Root mean square error of approximation (RMSEA)	0.052	0.065	0.065

* statistical significance at *p* < 0.05.

**Table 6:** The convergent and discriminant validity of the Malay version of the Shame and Stigma Scale (SSS-M).

Indicator Variables	Latent Variables	Standardized Loading	Square of Standardized Loading	Sum of Squared of Standardized Loading	Number of Indicators	AVE	Square Root of AVE	Inter-Construct Correlation
Item 2	SWA	0.729	0.531	2.784	5	0.557	0.746	SWA⇔SS = 0.708, SWA⇔R = 0.570
Item 3	SWA	0.683	0.466					
Item 5	SWA	0.731	0.534					
Item 6	SWA	0.827	0.684					
Item 8	SWA	0.754	0.569					
Item 9	SS	0.591	0.349	4.076	8	0.510	0.714	SS⇔SWA = 0.708, SS⇔R = 0.640
Item 10	SS	0.743	0.552					
Item 11	SS	0.798	0.637					
Item 12	SS	0.677	0.458					
Item 13	SS	0.610	0.372					
Item 14	SS	0.735	0.540					
Item 18	SS	0.781	0.610					
Item 19	SS	0.747	0.558					
Item 15	R	0.873	0.762	1.552	3	0.517	0.719	R⇔SWA = 0.570, R⇔SS = 0.640
Item 16	R	0.627	0.393					
Item 17	R	0.630	0.397					

AVE = average variance extracted, SWA= shame with appearance, SS = sense of stigma, R = regrets.

**Table 7:** The internal consistency, composite reliability index and test–retest reliability of the SSS-M.

Domains	Internal Consistency (Cronbach’s α)	Composite Reliability Index	Test–retest Reliability (Intraclass Correlation Coefficient)
Shame with appearance	0.747	0.862	0.789, *p* < 0.001 *
Sense of stigma	0.854	0.892	0.756, *p* < 0.001 *
Regret	0.685	0.758	0.843, *p* < 0.001 *
Total SSS	0.881	0.946	0.876, *p* < 0.001 *

* statistical significance at *p* < 0.05.

## Data Availability

The data presented in this study are available on request from the corresponding author. The data are not publicly available due to regulations of the ethics committees.
